# Religious Service Attendance, Allostatic Load, and Mortality Among Black Men

**DOI:** 10.1093/geroni/igab046.1633

**Published:** 2021-12-17

**Authors:** Marino Bruce, Bettina Beech, Dulcie Kermah, Gillian Marshall, Paul Archibald, Genee Smith, Roland Thorpe

**Affiliations:** 1 University of Houston, Houston, Texas, United States; 2 University of houston, Houston, Texas, United States; 3 Charles R. Drew University of Medicine and Science, Los Angeles, California, United States; 4 University of Washington, Tacoma, Washington, United States; 5 CUNY, Staten Island, New York, United States; 6 Johns Hopkins Bloomberg School of Public Health, Baltimore, Maryland, United States

## Abstract

Black men experience high levels of social and psychological stress and religion has been a coping strategy. The purpose of this study was to examine the association between religious service attendance and mortality among Black men. Data were drawn from the NHANES III (1988-1994) sample linked to the 2015 public use Mortality File. The analytic sample (n=2300) was restricted to Black men. All-cause mortality was the primary outcome and religious service attendance was the primary independent variable. Findings from Cox proportional hazards models indicated participants who attended at least once per week were 18% less likely to die than their peers who did not attend a religious service at all (fully adjusted HR 0.82; CI 0.68-0.99). The robust association between religious service attendance and mortality among Black men suggest that prospective studies are needed to further examine the influence of religion on health among this population.

